# mTORC1 signalling and protein synthesis are elevated in response to amino acids in human myotubes obtained from young, old, and old trained men

**DOI:** 10.1007/s11626-025-01041-2

**Published:** 2025-05-20

**Authors:** Stephanie D. Gagnon, Jiani Qian, Vladimir Belhac, Neil R. W. Martin

**Affiliations:** 1 School of Sport, Exercise and Health Sciences, Loughborough University, Loughborough, LE11 3TU UK; 2 Present Address: Raymond G. Perelman Center for Cellular and Molecular Therapeutics, The Children’s Hospital of Philadelphia, Philadelphia, PA USA

**Keywords:** Ageing, Anabolic resistance, MTOR, Myotubes, Exercise

## Abstract

Ageing and reduced levels of physical activity are associated with desensitisation of skeletal muscle to the anabolic effects of amino acids. In vitro studies have indicated that many properties of skeletal muscle tissue are retained in human myotubes, including metabolic alterations associated with exercise and disease. However, the interaction between ageing and physical activity on amino acid sensing and growth has not been explored in human myotubes in vitro. Muscle-derived cells were isolated from biopsies taken from eight young (Y: 23.4 ± 1.9 yr), six older (O: 72.5 ± 5.0 yr), and nine older exercise trained (OT: 71.0 ± 4.1 yr, *n* = 9) men, and myotube cultures were generated and investigated for growth parameters and amino acid induced changes in mTORC1 signalling and protein synthesis. Our results indicated that muscle cell fusion was similar between groups, but myotube diameter was lower in cultures derived from O individuals. Despite this, mTORC1 signalling, as indicated by immunoblots for phosphorylation of mTOR^Ser2448^, rpS6^Ser235/236^, and 4E-BP1^Thr37/46^ increased to a similar extent in response to amino acid availability in Y, O, and OT myotubes. Furthermore, measures of protein synthesis using the SUnSET assay were increased similarly between groups after the addition of amino acids. These data suggest that skeletal muscle desensitisation to amino acids with ageing is not observed in myotubes cultured in vitro, which could be reflective of the healthy individuals tested in our study or point towards the importance of the muscle niche in the impairments in muscle metabolism in ageing.

**Supplementary Information:**

The online version contains supplementary material available at 10.1007/s11626-025-01041-2.

## Introduction

Age-related loss of skeletal muscle size and strength (termed sarcopenia) leads to metabolic dysfunction, increased risk of falls and fractures, poor quality of life, and increased mortality (Cruz-Jentoft *et al*. 2019). Although the underpinning pathology is not clearly defined, older individuals display an attenuated rise in muscle protein synthesis (MPS) following elevations in amino acid availability compared to younger individuals (Wall *et al*. 2015). This so-called anabolic resistance to amino acids in aged muscle is associated with reduced phosphorylation of proteins in the mechanistic target of rapamycin complex 1 (mTORC1) signalling pathway (Guillet *et al*. 2004; Cuthbertson *et al*. 2005; Francaux *et al*. 2016), which is responsible for sensing amino acids and directing the cell towards anabolism and growth (Saxton and Sabatini 2017), and this desensitisation of amino acid sensing in skeletal muscle contributes to the development and progression of sarcopenia. However, recent studies of master athletes have demonstrated that high levels of exercise training can offset the typical skeletal muscle loss associated with ageing (Drey *et al*. 2016; Pollock *et al*. 2018; McKendry *et al*. 2020). By contrast, reductions in physical activity levels through either step reduction or limb immobilisation result in muscle loss, blunted elevations in MPS in response to amino acid availability, and delayed mTORC1 signalling (Glover *et al*. 2008; Breen *et al*. 2013). Collectively, these data suggest that reduced physical activity levels in older age may play a significant role in the development of sarcopenia and mTORC1 sensitivity to amino acids.

Skeletal muscle satellite cells can be isolated from human muscle tissue and differentiated in vitro to form myotube cultures, which are used to study fundamental muscle cell biology. Interestingly, numerous studies now indicate that human myotubes retain many of the metabolic characteristics of the donor muscle tissue. For example, human myotubes isolated from obese type II diabetics exhibit elevated inflammatory signalling and diminished insulin stimulated glucose uptake, thus mirroring the in vivo disease phenotype (Green *et al*. 2011). Furthermore, enhanced glucose and lipid oxidation can be detected in cultured human myotubes derived from individuals following a period of exercise training (Bourlier *et al*. 2013; Lund *et al*. 2017). Primary human myotubes derived from older individuals may also retain an aged phenotype. Several studies have demonstrated that myoblasts from older muscle have impaired differentiation and growth compared to younger cells (Pietrangelo *et al*. 2009; Beccafico *et al*. 2011; Brzeszczyńska *et al*. 2018; Bechshøft *et al*. 2019), mimicking the poor regenerative capacity of aged skeletal muscle tissue (Conboy *et al*. 2003). In addition, Bunprajun *et al*. (2013) found that myotubes from middle aged men exhibited insulin resistance, but that those from men who had undertaken lifelong exercise exhibited normal insulin sensitivity, suggesting that aged-myotubes may exhibit a memory of metabolism from the parent tissue. Recent evidence suggests that serum from older individuals can elicit atrophy in C2C12 myotubes and blunts the activation of some markers of mTORC1 signalling and the protein synthetic response following the addition of amino acids to the culture media (Allen *et al*. 2021). However, whether primary human myotubes derived from older men display an intrinsic impairment in mTORC1 signalling and MPS in response to amino acids, and how regular exercise training can influence these properties is unknown.

Thus, in the present study, we generated primary human myotube cultures from muscle biopsies taken from young, older, and older men who regularly undertake high levels of structured exercise with the aim of determining if mTORC1 signalling and protein synthesis differed between groups in response to altered amino acid availability. We hypothesised that myotubes from older men would display impaired growth and exhibit an attenuated mTORC1 and protein synthetic response compared to young myotubes and those derived from older exercise trained men.

## Materials and methods

### Participants and preliminary assessments

Eight young physically active (Y), six older (O), and nine older physically active (OT) men volunteered to participate in the present study and provided written informed consent. To be eligible for the study, young and older physically active men took part in at least 3 × 30 min of structured exercise (e.g. running, swimming, cycling, team sports) every week. All participants were non-smokers, normotensive (< 140/90) and non-obese (BMI < 30 kg/m^2^), and had no history of cardiovascular, metabolic, or haematological disorders or previous adverse responses to local anaesthetic. Participants had no disorders of blood coagulation and were not currently taking anti-inflammatory drugs or supplements. Ethical approval was granted from the Loughborough University research ethics committee (R17-P177), and the study conformed to the Declaration of Helsinki.

Participants visited the laboratory on three separate occasions separated by at least 48 h. During the first visit, blood pressure of the brachial artery was measured using an automated sphygmomanometer (Omron Healthcare, Milton Keynes, UK) with the subjects in a seated position, before height was measured using a stadiometer and measurements of body fat and body mass were assessed using bioelectrical impedance analysis (Seca, Hamburg, Germany). Thereafter, maximal handgrip strength was assessed using a handheld dynamometer (A5401 Digital Hand Grip Dynamometer, Takei, Japan), and maximal isometric quadriceps torque was assessed using isokinetic dynamometry (Cybex isokinetic dynamometer, HUMAC NORM, Stoughton, MA). For both handgrip strength and quadriceps torque, participants performed three maximal efforts on their dominant side and the average value was used for analysis. Lastly, participants completed an International Physical Activity Questionnaire (IPAQ) and were given a pedometer to wear for four consecutive days prior to their second laboratory visit. Participants returned to the laboratory on a second occasion for magnetic resonance imaging (MRI) of the dominant leg. The MRI was conducted by NHS radiographers based at the National Centre for Sport and Exercise Medicine at Loughborough University. MRI scans were performed and analysed as previously described (Balshaw *et al*. 2017).

### Muscle biopsy and cell isolation

Participants reported to the laboratory for their final visit following an overnight fast (> 10 h) and having refrained from any strenuous activity for the previous 24 h. Upon arrival, a 10-mL fasted venous blood sample was obtained and collected into EDTA and serum separator tubes (Sarstedt, Numbrecht, Germany). Subsequently, muscle biopsies of the vastus lateralis muscle on the dominant leg were obtained using the Bergstrom needle biopsy technique modified for use with suction. Muscle samples were blotted dry, dissected free of any obvious adipose or connective tissue, and then divided into two discrete portions. One portion was embedded in OCT mounting medium (Tissue-Tek, Sakura Finetek Europe, Alphen aan den Rijin, Netherlands), frozen in liquid-cooled isopentane and stored at − 80 °C until immunohistochemical analysis. The second portion (~ 100 mg) was submerged in growth media (GM) consisting of high-glucose DMEM (Sigma Aldrich, Gillingham, UK) supplemented with 20% foetal bovine serum (FBS; Pan Biotech, Wimborne, UK), and 100 U/mL penicillin and 100 µg/mL streptomycin (Gibco™, Fisher Scientific, Loughborough, UK). GM was also supplemented with 5 µg/mL amphotericin B (Gibco™, Fisher Scientific) at this stage. Muscle-derived cells (MDCs) were isolated using the explant technique as previously described (Lewis *et al*. 2000). In brief, muscle tissue was finely minced in GM and plated into gelatin-coated 25-cm^2^ flasks (Nunc™, Fisher Scientific), and incubated at 37 °C and 5% CO_2_. MDCs migrated from the tissue explants over the ensuing days and were detached from the surface of the flask using accutase® (Sigma Aldrich). The remaining explant tissue was discarded and the MDCs were sub-cultured for experimentation as described below.

### Cell culture and experimental details

MDCs were cultured in gelatin-coated 80-cm^2^ flasks (Nunc™, Fisher Scientific) in GM. Once cells reached 80% confluency, they were detached from the cell culture plastic using accutase® (Sigma Aldrich) and sub-cultured across serial passages to increase cell numbers. All experiments were conducted between passages 4 and 5, which was typically less than 30 days from the time of muscle biopsy.

In total, 50,000 cells were seeded into six-well plates and cultured in GM with media replenishment every 48 h. Once cells reached 90% confluence, myotube cultures were generated by replacing GM with differentiation media (DM) consisting of high-glucose DMEM supplemented with 2% horse serum (Sigma-Aldrich) and 100 U/mL penicillin and 100 µg/mL streptomycin (Gibco™, Fisher Scientific). Cells were maintained in DM for 5 d with the media completely replenished after 3 d and a sub-set of experimental cells were fixed for immunocytochemistry at this point. The remaining myotube cultures were washed 2 × with PBS and incubated for 6 h in amino acid free DMEM (US Biologicals, Salem, MA) in the absence of serum, and were subsequently re-fed with a combination of amino acids (see supplementary table 1) prepared in amino acid free media and of the same relative composition as a standard whey protein isolate drink typically consumed in human protein metabolism studies (UltraWhey 90; Volac, Hertfordshire, UK). For analysis of mTORC1 signalling, myotube cultures were lysed following 6 h of amino acid withdrawal, and after 0.5, 1, and 3 h of re-stimulation. For measurements of protein synthesis, we utilised the SUnSET assay which relies upon incorporation of the antibiotic and tyrosyl-tRNA analogue into nascent proteins and detection via immunoblotting (Schmidt *et al*. 2009). Puromycin was added to the culture media for the final 3 h of amino acid withdrawal or the entire 3 h of amino acid re-stimulation at which points cells were lysed for immunoblotting. We chose to include puromycin for the duration of the amino acid restimulation period to ensure that any temporal differences in protein synthesis rates between participants over the 3-h period were captured in the analysis.

### Blood analysis

EDTA blood tubes were immediately centrifuged at 1750 × *g* for 15 min at 4 °C to obtain plasma samples. Blood samples in serum separator tubes were left to clot at room temperature before centrifugation as described above. Plasma and serum samples were stored at − 20 °C until analysis. Plasma samples were analysed for fasting glucose concentrations with a semiautomated analyser (Pentra 400; Horiba Medical, Northampton, UK), and fasting serum insulin was determined using a commercially available ELISA kit (DRG Instruments GmbH, Marburg, Germany). The homeostatic model of insulin resistance was calculated as previously described (Hulston *et al*. 2018).

### Immunostaining

For muscle tissue immunohistochemistry, serial transverse (8 µm) muscle sections were cut, placed on poly-lysine coated microscope slides (Fisher Scientific), and fixed for 10 min in 3.7% formaldehyde. Tissue was then blocked for 1 h at room temperature in tris buffered saline (TBS) containing 5% goat serum (Abcam, Cambridge, UK), 2% bovine serum albumin (BSA, Fisher Scientific), and 0.2% triton X-100 (Sigma Aldrich). Following 3 × TBS washes, sections were incubated for 1 h with myosin heavy chain type I primary antibody (A4.951 was deposited to the DSHB by Blau, H.M.) diluted in blocking solution. Slides were washed 3 × in TBS before incubation for 2 h with goat anti-mouse AlexaFluor® 488 conjugated IgG secondary antibody (Invitrogen, ThermoFisher, Paisley, UK) and AlexaFluor® 350 conjugated wheat germ agglutinin (Invitrogen), diluted in blocking solution. Coverslips were mounted to slides using Fluoromount™ aqueous mounting medium (Sigma Aldrich).

For muscle cell immunocytochemistry, cells were washed twice in PBS, fixed in ice-cold methanol and acetone, and then blocked for 1 h at room temperature in TBS containing 0.2% Triton-X 100 (Sigma Aldrich) and 5% normal goat serum (Abcam). After 3 × TBS washes, cells were incubated for 2 h with monoclonal desmin antibody (Clone D33, Dako, Clostrup, Denmark) diluted 1:100 in TBS containing 0.2% Triton X-100 and 2% normal goat serum. Following three further washes, cells were incubated for 2 h with AlexaFluor® 488 conjugated goat anti-mouse IgG1 secondary antibody (Invitrogen) diluted 1:200 in TBS containing 0.2% Triton X-100 and 2% normal goat serum. 4′,6-Diamidino-2-phenylindole (DAPI) was included in the secondary antibody solution at a dilution of 1:1000, to counterstain nuclei. Coverslips were mounted onto glass microscope slides using Fluoromount™.

All images were captured using a Leica DM2500 fluorescent microscope (Leica, Wetzlar, Germany). Muscle fibre cross-sectional area and myotube diameter were measured using Fiji image analysis software (Schindelin *et al*. 2012). Cross-sectional areas were defined by the wheat germ agglutinin stained muscle fibre boundary. Fusion index was calculated as the number of desmin-positive cells in a myotube as a percentage of the total number of desmin-positive cells per image.

### Immunoblotting

Myotube cultures were washed twice with ice cold PBS and lysed in 200 µL of RIPA buffer (Fisher Scientific) containing a protease and phosphatase inhibitors. In total, 10 µg of protein was loaded into 4–15% Mini-PROTEAN precast gels (Bio-Rad, Hemel Hempstead, UK) and separated by SDS-PAGE at 150 V. The separated proteins were transferred onto 0.2-µm polyvinylidene difluoride (PVDF) membranes (Bio-Rad) at a constant current of 0.25A, and were then washed 3 × in TBS containing 0.1% Tween20 (TBST) and blocked in either 5% non-fat milk (Bio-Rad) or 5% bovine serum albumin (BSA; Fisher Scientific) diluted in TBST for 60 min at 4 °C. After three further TBST washes, the membranes were incubated overnight at 4 °C with primary antibodies against phospho-mTOR^Ser2448^ (1:2000 in 2% BSA; Cell Signaling Technology, Danvers, MA, #5536), phospho-rpS6^Ser235/236^ (1:2000 in 2% milk; Cell Signaling Technology, #2211), phospho-4E-BP1^Thr37/46^ (1:2000 in 2% milk; Cell Signaling Technology, #2855), or anti-puromycin (1:5000 in 1% BSA; Merck, Watford, UK, #MABE343). Membranes were then washed three times with TBST and incubated for 1 h at room temperature in anti-rabbit IgG HRP-conjugated secondary antibody (Cell Signaling Technology) at a concentration of 1:2000 in either 2% milk or 2% BSA, consistent with the primary antibody dilution, before detection using chemiluminescence. Signals were captured and quantified within the linear range of detection on the Chemidoc XRS system (Bio-Rad) using Quantity One image software (Version 4.6.8, Bio-Rad). Protein phosphorylation was normalised to protein loading through Coomassie blue staining and subsequent selection of a protein band (Bass *et al*. 2017).

### Statistical analysis

All statistical tests were performed using IBM SPSS Statistics version 29 (IBM SPSS Inc., Chicago, IL). Data were checked for normality using Shapiro–Wilk tests, and for homogeneity of variance using Levene’s test or Mauchly’s test of Sphericity as appropriate. Participant characteristics and myotube morphology were assessed using one-way ANOVA with Tukey post hoc analyses when appropriate, or Kruskal–Wallis tests with pairwise Mann–Whitney tests where data was not normally distributed. mTORC1 signalling and puromycin incorporation analyses were assessed using mixed-measures ANOVA with multiple comparisons undertaken with Bonferroni adjustment as required. All data are presented as mean ± standard deviation (SD) unless stated otherwise, and statistical significance was set at *p* < 0.05.

## Results

### Participant characteristics

Participant characteristics are shown in Table 1. Daily step count was lower in the O group compared to both the Y and OT groups (*p* < 0.05 and *p* < 0.01 respectively) with no difference between Y and OT groups (*p* = 0.904). IPAQ scores mirrored this pattern of physical activity, with O group exhibiting lower MET/min compared with both the Y and OT groups respectively (both *p* < 0.01), with no difference between Y and OT groups (*p* = 0.911). This suggests that overall physical activity was equivalent between Y and OT groups, and lower in the O group. There was no difference in BMI between groups (*p* = 0.355); however, percentage body fat was lower in Y compared to both O (*p* < 0.001) and OT (*p* < 0.01) groups. Fasting plasma glucose concentration was lower in Y compared to O and OT groups (both *p* < 0.05), but no differences existing between groups for fasting serum insulin (*p* = 0.28) or HOMA-IR (*p* = 0.279) measurements.

**Table 1.** Characteristics of the participants from which myotube cultures were derived

	Young (*n* = 8)	Old (*n* = 6)	Old trained (*n* = 9)
Anthropometrics			
Age (yr)	23.4 ± 1.9	72.5 ± 5.0^a^	71.0 ± 4.1^a^
BMI (kg/m^2^)	24.9 ± 2.9	25.7 ± 2.6	23.9 ± 1.6
Body fat (%)	15.7 ± 3.5	27.1 ± 4.6^a^	23.1 ± 3.9^a^
Blood analyses			
Fasting plasma glucose (mmol/L)	4.6 ± 0.6	5.5 ± 0.6^c^	5.4 ± 0.6^c^
Fasting serum insulin (µIU/mL)	9.0 ± 5.6	11 ± 7.2	6.6 ± 2.8
HOMA-IR	1.9 ± 1.3	2.8 ± 2.2	1.6 ± 0.7
Physical activity			
Steps/day	9254 ± 2206	4967 ± 2774^c,e^	9886 ± 3004
IPAQ (Mets/min/week)	7003.5 ± 2512.7	2753.0 ± 1268.1^b,d^	7452.2 ± 2455.4
Muscle and strength characteristics			
Quadriceps volume (cm^3^)	2261.2 ± 347.7^$^	1537.0 ± 173.0^$b^	1665.2 ± 253.1^c^
Type 1 (%)	39.5 ± 14.8	45.5 ± 20.6	61.0 ± 11.3^c^
Type I CSA (µm^2^)	4589.4 ± 1137.8	4764.2 ± 1773.9	5196.9 ± 825.6
Type II (%)	60.5 ± 14.8	54.5 ± 20.6	39.0 ± 11.3^c^
Type II CSA (µm^2^)	5594.9 ± 1803.0	3871.8 ± 489.8^c^	4330.8 ± 454.6
Peak hand grip strength (kg)	47.0 ± 6.5	39.8 ± 2.9^c^	39.6 ± 3.3^b^
Peak quadriceps torque (Nm)	280.5 ± 66.6	180.2 ± 22.3^b^	180.1 ± 41.4^b^

*BMI*, body mass index; *IPAQ*, International Physical Activity Questionnaire; *HGS*, peak hand grip strength; *CSA*, cross-sectional area; *HOMA-IR*, homeostatic model assessment for insulin resistance

^$^*n* = 7 and *n* = 5 for this analysis in young and old groups respectively

^a^Significantly different from young (*p* < 0.001)

^b^Significantly different from young (*p* < 0.01)

^c^Significantly different from young (*p* < 0.05)

^d^Significantly different from old trained (*p* < 0.01)

^e^Significantly different from old trained (*p* < 0.05)

Based on MRI scans of the dominant leg, quadriceps volume was on average 47% greater in Y compared with O (*p* < 0.01) and 36% greater than OT (*p* < 0.05). Furthermore, type II fibre CSA was significantly greater in Y compared to the O group (*p* < 0.05), with no difference between Y and OT groups (*p* = 0.08). There was no difference in type I CSA between groups (*p* = 0.42). There was a greater proportion of type I, and fewer type II fibres in OT muscle biopsies compared to Y (*p* < 0.05), with no other difference between groups. Finally, hand grip strength (*p* < 0.05 and *p* < 0.01) and peak quadricep torque (both *p* < 0.01) were greater in Y than both O and OT groups respectively.

### Myotube morphology

**Figure 1.**
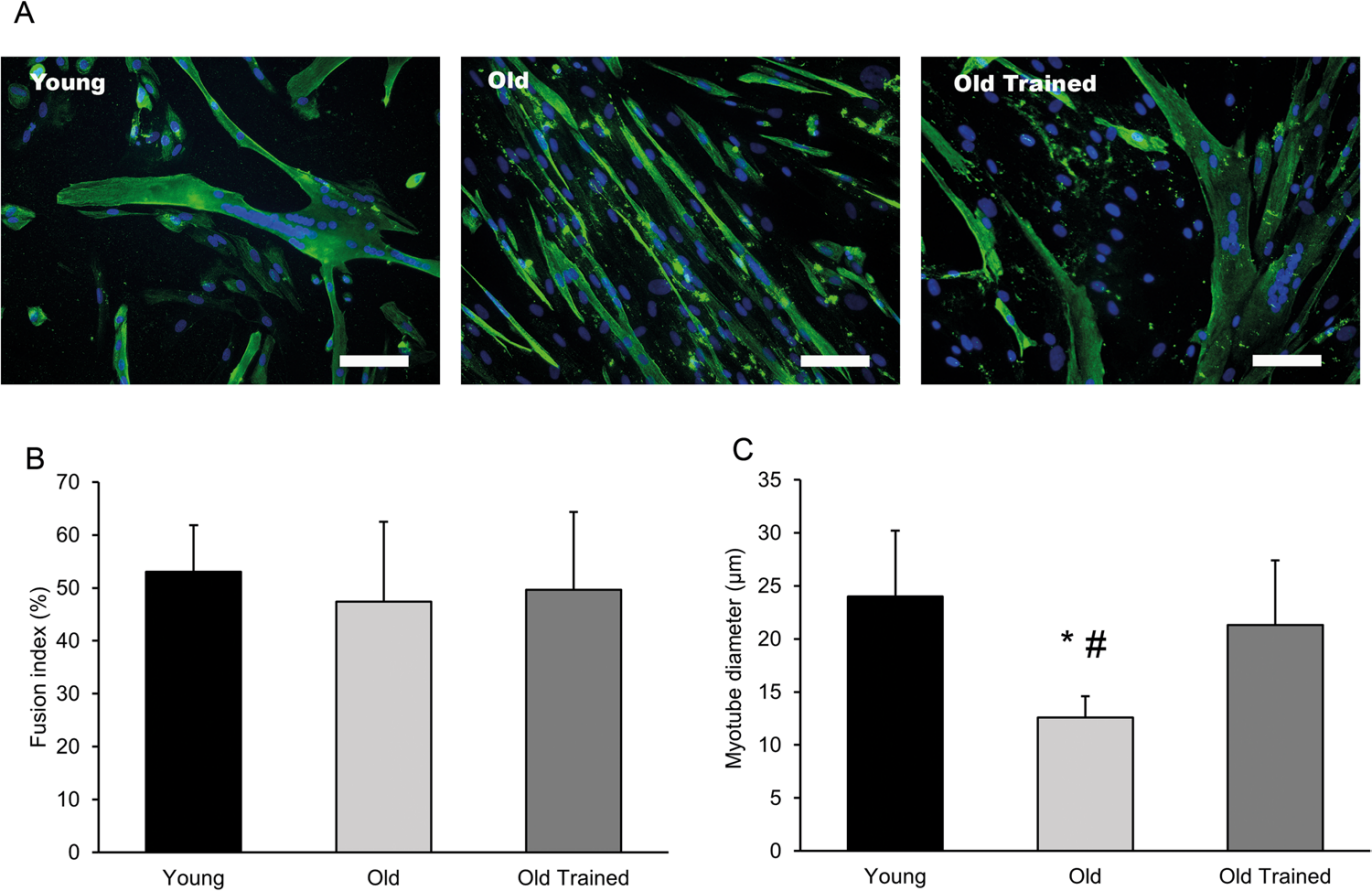
The effect of ageing and exercise training on myotube morphology. Primary human myotube cultures were generated from muscle derived cells isolated from muscle biopsies of young (18–25 y), old (65–80 y, undertaking no structured exercise), and old trained men (65–80 y, undertaking at least 3 structured exercise sessions per wk). (*A*) Muscle cells were cultured in low serum media for 5 d to induce differentiation, prior to fixation and subsequent staining for desmin (*green*) and nuclei (*blue*). (*B*) Fusion index, calculated as the percentage of desmin-positive cells incorporated into myotubes was not different between groups. (*C*) Myotube diameter was significantly lower in cultures from old individuals compared to those derived from young and old trained men. Data are mean ± SD from *n* = 8 young, 6 old, and 9 old trained cultures. * indicates statistically lower than young (*p* < 0.01), # indicates statistically lower than old trained (*p* < 0.05). *Scale bar* is 100 µm.

To investigate the effects of ageing and physical activity status on muscle precursor cell differentiation, cells were fixed and stained for the muscle specific intermediate filament protein, desmin, after 5 d in low serum media (Fig. 1*A*). There was no difference in the percentage of desmin-positive cells isolated from muscle biopsies between groups (*p* = 0.392) with cell cultures consisting of 68.5 ± 13.6, 63.6 ± 15.8, and 73.4 ± 11.3% desmin-positive cells in Y, O, and OA groups respectively. Based on previous literature, we assumed that cells which did not stain for desmin were fibroblasts (Agley *et al*. 2013; Mackey *et al*. 2017), and deliberately maintained them in our myotube cultures to recapitulate the native tissue where they influence muscle regeneration and maturation (Murphy *et al*. 2011; Mackey *et al*. 2017). The ability of the desmin-positive cells to fuse into multinucleate myotubes in vitro appeared to be comparable between groups as fusion index was not different (*p* = 0.721, Fig. 1*B*). However, myotubes derived from the O group exhibited a 47% smaller myotube diameter compared to Y (*p* < 0.01) and a 41% smaller diameter compared to myotubes derived from OT (*p* < 0.05 respectively) indicating potential impairments in the ability of these myotubes to undertake further growth (Fig. 1*C*).

### mTORC1 signalling and protein synthesis

**Figure 2.**
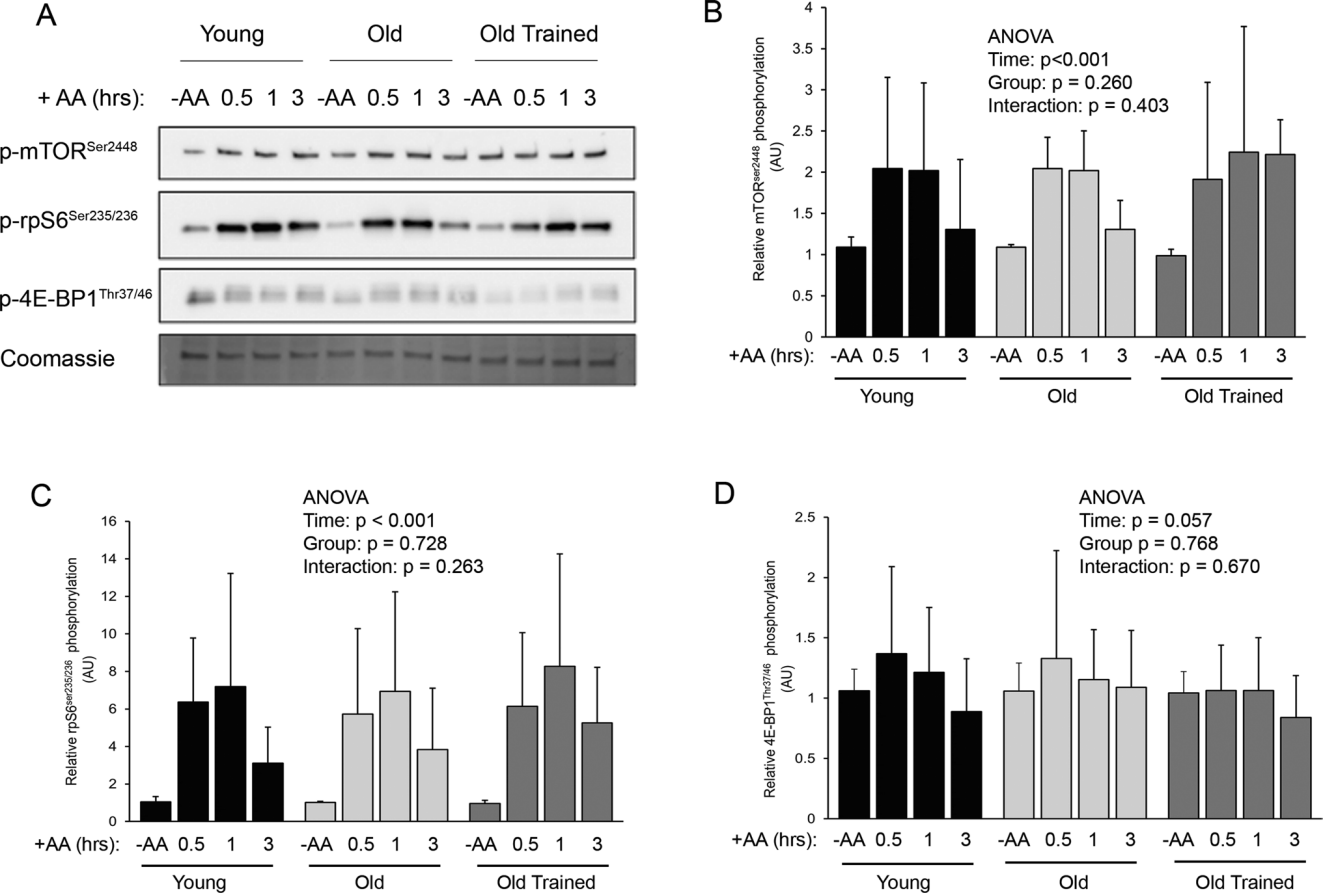
Time-dependent changes in mTORC1 signalling in myotube cultures derived from young, old, and old trained individuals in response to amino acid availability. (*A*) Immunoblots for p-mTOR^ser2448^, p-rpS6^ser235/236^, and p-4E-BP1^Thr37/46^. (*B*) Quantification of mTOR^ser2448^. (*C*) Quantification of rpS6^ser235/236^. (*D*) Quantification of 4E-BP1^Thr37/46^. Data are mean ± SD from *n* = 8 young, 6 old, and 9 old trained myotube cultures.

The sensitivity of the anabolic mTORC1 pathway to nutrient availability was assessed by measuring phosphorylation of mTOR^ser2448^ and its downstream substrates ribosomal protein S6^Ser235/236^ (rpS6 ^Ser235/236^) and 4E-BP1^Thr37/46^ in the absence or presence of amino acids. Our preliminary data indicated that in accordance with previous literature in C2C12 cells, human primary myotube cultures showed robust stimulation of mTORC1 signalling by leucine, whereas other amino acids induce a negligible response (Figure S1*a*). To thus ensure adequate mTORC1 stimulation, as well as sufficient substrate for protein synthesis, myotube cultures were fed a combination of amino acids at a ratio equivalent to a whey protein supplement, and relative to 1 mM leucine (Figure S1*b*). Both mTOR ^ser2448^ and ribosomal S6 ^Ser235/236^ phosphorylation increased in response to amino acid availability (Fig. 2) with no difference observed between groups or interaction effects (all *p* > 0.05). 4E-BP1^Thr37/46^ phosphorylation showed a trend towards temporal changes with the introduction of amino acids (*p* = 0.057), with no differences between groups or interaction effects observed. Collectively, this data demonstrates that myotube cultures derived from Y, O, and OT groups display similar mTORC1 sensitivity to amino acids.

**Figure 3.**
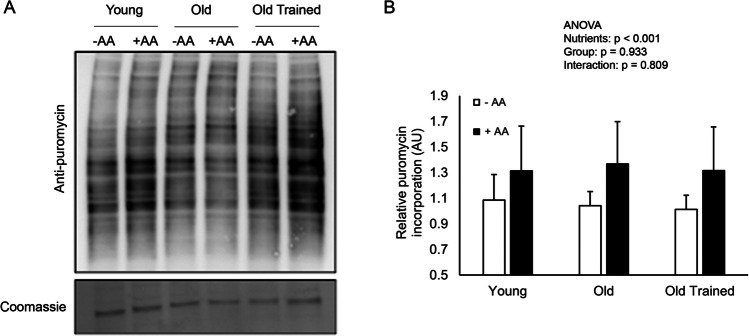
The effect of amino acid availability on protein synthesis in myotube cultures derived from young, old, and old trained individuals. (*A*) Immunoblot and (*B*) quantification of puromycin incorporation into nascent proteins in myotube cultures following puromycin incubation in the final 3 h of a 6-h amino acid deprivation period, or the entire 3-h re-feeding period. Data are means ± SD from *n* = 6 young, 6 old, and 8 old trained myotube cultures.

Protein synthesis was assessed via puromycin incorporation into nascent proteins using the SUnSET method (Fig. 3). Puromycin incorporation increased by 21, 30, and 31% in Y, O, and OT myotube cultures respectively following the addition of amino acids (*p* < 0.001), demonstrating a stimulation of protein synthesis by amino acids; however, the extent of this increase was not different between groups (*p* = 0.933).

## Discussion

Ageing and reduced physical activity levels can desensitise skeletal muscle to amino acid availability through aberrant mTORC1 signalling, resulting in impaired rates of protein synthesis and muscle wasting. Whilst in vitro cultures of primary human myotubes have been shown to retain characteristics of donor muscles such as impaired muscle regeneration associated with ageing (Pietrangelo *et al*. 2009; Beccafico *et al*. 2011; Brzeszczyńska *et al*. 2018; Bechshøft *et al*. 2019) and enhanced substrate oxidation following exercise training (Bourlier *et al*. 2013; Lund *et al*. 2017), the effects of ageing and physical activity levels on amino acid induced mTORC1 signalling and protein synthesis in human myotubes are unknown. The present data demonstrate that muscle cells obtained from older people with low levels of physical activity differentiate into smaller sized myotubes; however, the mTORC1 and protein synthetic responses to amino acid availability were equivalent in myotubes derived from old (O), old trained (OT), and younger (Y) individuals.

Morphologically, we observed smaller myotubes in cultures of O muscle cells compared with Y and OT groups, but no difference in the fusion capabilities between groups. It is often reported that muscle cells from older individuals exhibit a reduced ability to differentiate in vitro (Pietrangelo *et al*. 2009; Beccafico *et al*. 2011; Bechshøft *et al*. 2019); however, others have observed a normal capacity for fusion in aged myoblast in accordance with our own findings (Conboy *et al*. 2003; Alsharidah *et al*. 2013) and these inconsistencies in the literature cannot be accounted for by the myogenic purity of the starting cell population (Alsharidah *et al*. 2013; Bechshøft *et al*. 2019). The extent of myoblast differentiation may be related to the induction of a senescent molecular programme, since senescent primary human myoblasts have impaired fusion compared to proliferative cells (Alsharidah *et al*. 2013; Francis *et al*. 2022), and increased markers of cell senescence have been observed in aged muscle cells exhibiting low levels of fusion (Bechshøft *et al*. 2019). Whilst we did not measure markers of senescence in our cell cultures, the muscle cells derived from older individuals were still proliferative at passages beyond those used in the present study and so it is likely that senescence was not reached. Importantly, although myotubes derived from O cells were smaller than those from Y, OT myotubes were not, suggesting that exercise training has positive effects on muscle satellite cells that are retained when isolated and cultured in vitro*.* It is likely that these effects are related to the pathways regulating cell growth such as mTORC1 and whilst we did not find changes in mTORC1 signalling between groups, future studies could seek to profile the expression of molecular regulators of muscle anabolism and catabolism in these cells.

The present data show that myotube cultures derived from Y, O, and OT groups exhibit similar increases in mTORC1 signalling and protein synthesis in response to amino acid availability. This contrasts with human in vivo studies which have observed skeletal muscle desensitisation or “anabolic resistance” to amino acid provision with ageing (Volpi *et al*. 2000; Guillet *et al*. 2004; Cuthbertson *et al*. 2005; Wall *et al*. 2015; Francaux *et al*. 2016; Smeuninx *et al*. 2017) and reduced physical activity levels (Glover *et al*. 2008; Drummond *et al*. 2012; Breen *et al*. 2013; Wall *et al*. 2016) and suggests that these altered metabolic properties of aged skeletal muscle are possibly more associated with amino acid delivery to the muscle (Moro *et al*. 2016) or the local muscle environment than intrinsic to the muscle cells themselves. In support of this, Allen *et al*. (2021) recently demonstrated that exposure of C2C12 myotubes to serum obtained from older people blunted the increase in protein synthesis and several mTORC1-related proteins in response to amino acid provision, compared with myotubes treated with young serum. Furthermore, in both rodents and humans, age-related attenuation of mTORC1 and protein synthesis in response to nutrients are associated with systemic inflammation (Balage *et al*. 2010; Draganidis *et al*. 2021), and as such removing cells from the in vivo environment and culturing them in vitro may account for the normal anabolic sensitivity observed in our experiments. In future studies, it would certainly be interesting to culture primary human myotubes with autologous serum obtained in both fasted and fed states to better mimic the extracellular niche.

If the local muscle environment drives age-related amino acid insensitivity, then the use of tissue explants to isolate muscle-derived cells in the present set of experiments may have further inhibited our ability to observe age/exercise-related effects. Although the explant method is commonly used (Merrick *et al*. 2010) and encourages satellite cell activation and expansion in a more ecologically valid manner than enzymatic digests, in our hands the time from muscle biopsy to experimentation occasionally exceeded 1 mo. As such, it is possible that the acute effects of the local muscle environment on metabolism may be lost over this extended time in an artificial in vitro environment. Indeed, studies that have demonstrated retention of in vivo characteristics, such as insulin resistance associated with metabolic diseases or enhancements in glucose and fat metabolism with exercise, have consistently employed the use of enzymatic digests to isolate muscle cells from the biopsy tissue (Ukropcova *et al*. 2005; Bourlier *et al*. 2013; Bunprajun *et al*. 2013; Green *et al*. 2013; Heden *et al*. 2017; Lund *et al*. 2017) which can be accomplished within 60 min. As such, we cannot discount the possibility that we would have observed different results if the time between obtaining the muscle biopsy and conducting the cell culture experiments was reduced.

Human studies have suggested that age-related anabolic resistance to amino acids can be somewhat overcome by increased amino acid abundance (Moore *et al*. 2015), and anabolic resistance is most commonly observed at lower to moderate amino acid intakes. In the present study, we treated cells with a combination of amino acids similar to that used in human metabolism studies made relative to 1 mM L-leucine. This decision was based on our pilot experiments illustrating that leucine was stimulatory to mTORC1 whereas other amino acids showed little effect, but nonetheless are important substrates for protein synthesis. However, in adopting this approach, it is possible that we saturated the mTORC1 and protein synthetic responses in Y, O, and OT myotube cultures which would help account for the lack of group differences observed in our experiments. Other studies have observed stimulation of protein synthesis in myotube cultures with leucine alone (Lawrence *et al*. 2018; Osburn *et al*. 2021), and therefore employing a dose–response study on the effect of leucine on mTORC1 protein synthesis in Y, O, and OT myotubes may have allowed for a better understanding of anabolic sensitivity to amino acids to be garnered.

Another explanation for our in vitro results is that the participants in the O group may not have exhibited anabolic resistance to amino acids in vivo. Whilst the concept of anabolic resistance to amino acids in ageing is well established, several studies have found no evidence for it (Shad *et al*. 2016), which may be related to factors such as source and total amount of amino acids provided in these studies, as well as habitual physical activity levels of the participants. Indeed, we hypothesised that reduced sensitivity of muscle anabolism to amino acids in myotube cultures would be related to physical activity levels rather than ageing per se; however, the minimum physical activity levels required to maintain anabolic sensitivity to nutrients are unknown. For example, step reduction to < 1500 steps/day (Breen *et al*. 2013) or bed rest/immobilisation (Glover *et al*. 2008; Drummond *et al*. 2012; Wall *et al*. 2016) induces anabolic resistance to nutrients, whereas the participants in the O group of our study recorded approximately 5000 steps/day on average, and exhibited muscle strength and size that were indicative of healthy ageing. Recently, Horwath *et al*. (2024) found no evidence for anabolic resistance to amino acids in older men who were lean and physically active (but not well-trained/athletes) which supports the concept that habitual physical activity can counteract anabolic resistance, and this in turn may account for our in vitro findings (Horwath *et al*. 2024).

Finally, it is of note that the equivalent levels of protein synthesis and mTORC1 signalling between groups in response to amino acid stimulation seems at odds with the morphological data showing smaller myotube size in O compared with Y and OT cultures. There are several reasons that could account for this discrepancy. Firstly, although amino acid stimulation of protein synthesis was not different between groups, basal levels of protein synthesis might be altered between groups, and differences may have been apparent at time points prior to the onset of experimentation in the present study (e.g. during myogenesis). Although studies in humans do not support this idea (Cuthbertson *et al*. 2005; Drummond *et al*. 2008), this remains to be tested in human myotubes. Secondly, we cannot discount the possibility that myotubes in the O group exhibited impaired anabolic responses to amino acids that were masked by normal changes in these parameters in the non-myogenic (fibroblasts) and/or unfused desmin-positive cells within the cultures. Immunocytochemical detection of mTORC1 signalling and puromycin incorporation would be required in future studies to determine if this is the case. Finally, the smaller myotubes in the O group could be accounted for by elevated proteolytic degradation (e.g. ubiquitin proteasome or autophagy lysosome systems), measures of which were beyond the scope of this study.

## Conclusions

In conclusion, we have shown equivalent increases in mTORC1 signalling and protein synthesis in myotube cultures derived from young, old, and old trained men in response to amino acid stimulation in vitro. Future studies should seek to understand if the method of muscle cell isolation and/or the time in culture can influence measures of metabolism in human primary myotubes. Furthermore, additional clarity on the aetiology of anabolic resistance and the role that physical activity plays in its prognosis should be determined, and to this end human primary myotubes may be a useful tool in the future.

## Supplementary Information

Below is the link to the electronic supplementary material.

Supplementary file1 (DOCX 21 KB)

Supplementary file2 (PNG 423 KB)

## Data Availability

The data that support the findings of this study are available from the authors upon request.

## Declarations

### Disclaimer

The views expressed are those of the authors and not necessarily those of the NHS, the NIHR, or the Department of Health and Social Care.
