# Supplementary material for: mTORC1 signalling and protein synthesis are elevated in response to amino acids in human myotubes obtained from young, old, and old trained men
Source: In Vitro Cell Dev Biol Anim. 2025 May 20;62(2):139–48. doi: 10.1007/s11626-025-01041-2 (PMC12975814; doi:10.1007/s11626-025-01041-2)
Supplement: Supplementary file 1 — Supplementary file1 (DOCX 21 KB) [file 11626_2025_1041_MOESM1_ESM.docx]

**Supplementary information**

**Table S1.** Amino acids and their respective concentrations used to stimulate mTORC1 and protein synthesis in myotube cultures.

| **Amino Acid** | **Final concentration (mM)** |
| --- | --- |
| Alanine | 0.5 |
| Arginine | 0.2 |
| Aspartic Acid | 1.0 |
| Cysteine | 0.2 |
| Glutamic Acid | 1.7 |
| Glycine | 0.1 |
| Histidine | 0.2 |
| Isoleucine | 0.6 |
| Leucine | 1.0 |
| Lysine | 0.9 |
| Methionine | 0.2 |
| Phenylalanine | 0.3 |
| Proline | 0.5 |
| Serine | 0.4 |
| Threonine | 0.6 |
| Tryptophan | 0.1 |
| Tyrosine | 0.2 |
| Valine | 0.6 |

**Figure S1.** The effect of amino acids on mTORC1 signalling and protein synthesis in primary human myotubes. A) Myotubes were starved of serum and amino acids for 6 hours before being treated with 1mM of individual essential amino acids and immunoblotting for rpS6^ser235/236^ phosphorylation as a marker of mTORC1 activity. Arginine was also included in the experiment since previous reports have indicated that it is stimulatory to mTORC1 signalling (Chantranupong et al. 2016)). Dotted line indicates levels of rpS6^ser235/236^ in nutrient restricted/starved myotubes. B) Measures of protein synthesis conducted in human myotubes cultures (25 yrs old) receiving normal differentiation media (DMEM + 2% horse serum), amino acid and serum free DMEM, or amino acid and serum free DMEM supplemented with a combination of amino acids as described in Table S1. DMEM = Dulbecco’s modified Eagles medium; AAs = Amino acids.
