# Supplementary figures and images for: mTORC1 signalling and protein synthesis are elevated in response to amino acids in human myotubes obtained from young, old, and old trained men
Source: In Vitro Cell Dev Biol Anim. 2025 May 20;62(2):139–48. doi: 10.1007/s11626-025-01041-2 (PMC12975814; doi:10.1007/s11626-025-01041-2)

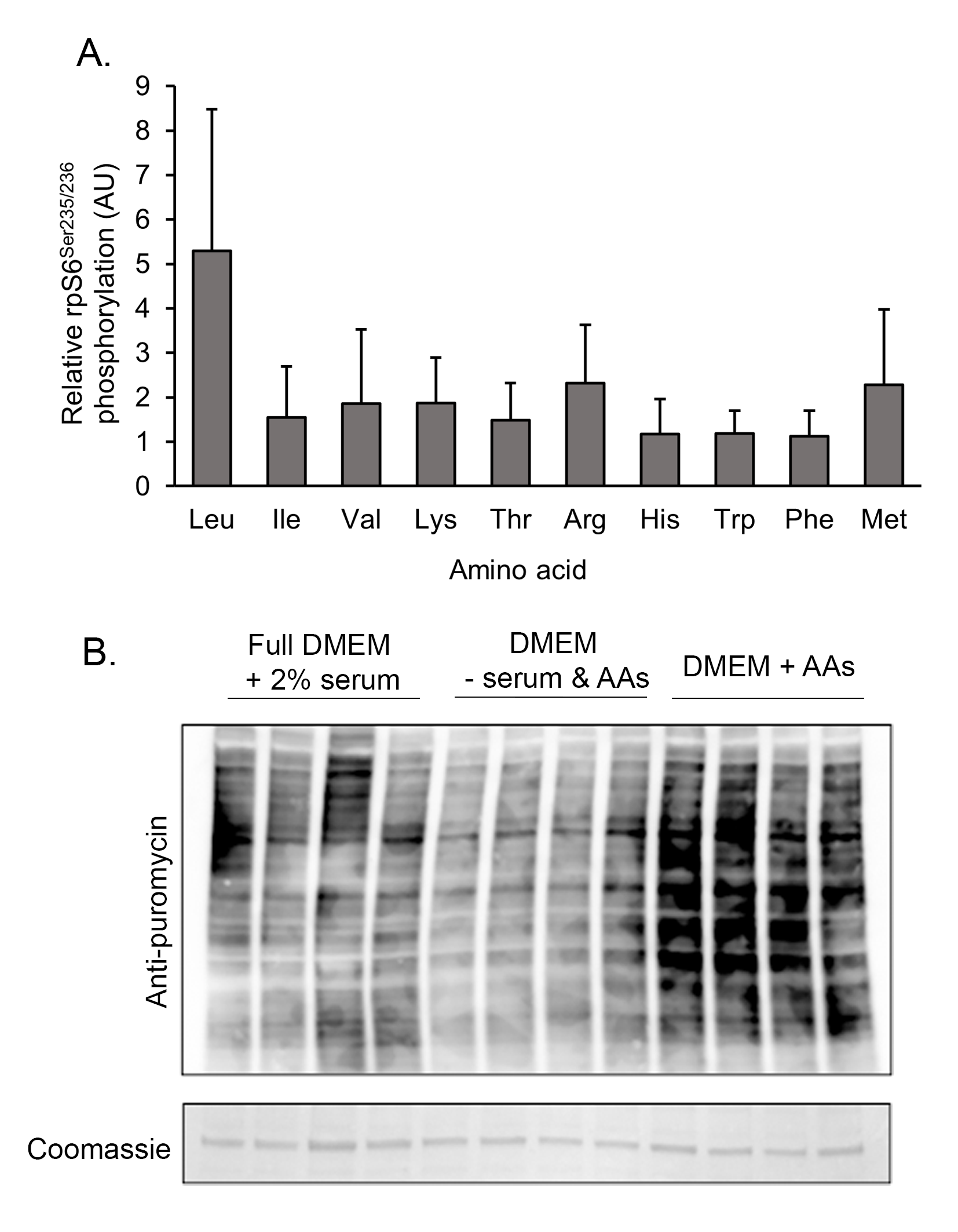

Supplement: Supplementary file 2 — Supplementary file2 (PNG 423 KB) [file 11626_2025_1041_MOESM2_ESM.png]
